# Molecular Cloning of Novel-Type Phospho*enol*pyruvate Carboxylase Isoforms in Pitaya (*Hylocereus undatus*)

**DOI:** 10.3390/plants9091241

**Published:** 2020-09-21

**Authors:** Keiichi Nomura, Yuho Sakurai, Mayu Dozono

**Affiliations:** 1 Graduate School of Agricultural Science, Kobe University, 1-1 Rokkodai-cho, Nada-ku, Kobe, Hyogo 657-8501, Japan; 185a316a@stu.kobe-u.ac.jp; 2 Faculty of Agricultural Science, Kobe University, 1-1 Rokkodai-cho, Nada-ku, Kobe, Hyogo 657-8501, Japan; 1756226a@stu.kobe-u.ac.jp

**Keywords:** CAM, isoform, phospho*enol*pyruvate carboxylase, pitaya (*Hylocereus undatus*)

## Abstract

Phospho*enol*pyruvate carboxylase (PEPC) is an important enzyme involved in the initial CO_2_ fixation of crassulacean acid metabolism (CAM) photosynthesis. To understand the cultivation characteristics of a CAM plant pitaya, it is necessary to clarify the characteristics of PEPC in this species. Here, we cloned three PEPC cDNAs in pitaya, *HuPPC1*, *HuPPC2*, and *HuPPC3*, which encode 942, 934, and 966 amino acid residues, respectively. Phylogenetic analysis indicated that these PEPC belonged to plant-type PEPC (PTPC), although HuPPC1 and HuPPC2 have no Ser-phosphorylation motif in N-terminal region, which is a crucial regulation site in PTPC and contributes to CAM periodicity. HuPPC1 and HuPPC2 phylogenetically unique to the Cactaceae family, whereas HuPPC3 was included in a CAM clade. Two isoforms were partially purified at the protein level and were assigned as HuPPC2 and HuPPC3 using MASCOT analysis. The most distinct difference in enzymatic properties between the two was sensitivity to malate and aspartate, both of which are allosteric inhibitors of PEPC. With 2 mM malate, HuPPC3 was inhibited to 10% of the initial activity, whereas HuPPC2 activity was maintained at 70%. Aspartate inhibited HuPPC3 activity by approximately 50% at 5 mM; however, such inhibition was not observed for HuPPC2 at 10 mM. These results suggest that HuPPC3 corresponds to a general CAM-related PEPC, whereas HuPPC1 and HuPPC2 are related to carbon and/or nitrogen metabolism, with a characteristic regulation mechanism similar to those of Cactaceae plants.

## 1. Introduction

Phospho*enol*pyruvate (PEP) carboxylase (PEPC, EC 4.1.1.31) is an enzyme that catalyzes the carboxylation of PEP, followed by the release of oxaloacetate (OAA) and inorganic phosphate (Pi). PEPC is widely distributed in plant kingdom and some microorganisms. In plants, there are two types of PEPC: plant-type PEPC (PTPC) and bacterial-type PEPC (BTPC) [1]. The former, usually called Class-1, PEPC consists of a homotetramer of a ~110 kDa subunit. The latter, which has a larger molecular mass (~117 kDa) and forms a hetero-octamer, is called Class-2 PEPC and consists of Class-1 PEPC and four BTPC subunits. PTPCs can be broadly categorized further into two groups according to their functions: photosynthetic and nonphotosynthetic isozymes. The main function of nonphotosynthetic PEPC was thought to be its contribution to an anaplerotic reaction [1,2]. Additionally, nonphotosynthetic PEPC also participates in many metabolic processes, such as nitrogen metabolism [3,4,5], redox balance [6], stress response [7,8]. Because of its diversity, PEPC consists of a multigene family, and its activity is regulated by some metabolites that behave as allosteric inhibitors or effectors. The typical regulation mechanism underlying PEPC activity is caused by phosphorylation at a serine (Ser) residue in the N-terminal extension region [9]. The sensitivity to allosteric inhibitors such as malate and aspartate (Asp) is decreased by phosphorylation and restored by dephosphorylation [10,11,12]. The phosphorylation and dephosphorylation are also important for the regulation of photosynthetic PEPCs, especially in crassulacean acid metabolism (CAM) plants. In CAM photosynthesis, CO_2_ is fixed to PEP through the action of PEPC, and the generated OAA is reduced to malate through the action of malate dehydrogenase; subsequently, malate is transferred to and stored in the vacuole. These CO_2_-concentrating reactions proceed during the night, CO_2_ is released from the stored malate and is assimilated during the day. In this sequential process, the sensitivity of PEPC to malate is controlled by phosphorylation, catalyzed by PEP carboxylase kinase (PPCK), and dephosphorylation through the catalytic action of protein phosphatase 2A (PP2A) [11]. Phosphorylated PEPC exhibits low malate sensitivity, resulting in high efficiency to CO_2_ fixation, whereas dephosphorylated PEPC is strongly inhibited by malate to avoid a futile cycle. Therefore, the major factor for this circadian rhythm may not be expression of PEPC, but of PPCK [13]. All PTPCs, C_3_ and/or nonphotosynthetic, C_4_ and CAM, have the Ser-phosphorylation motif, and both C_4_—and CAM-related PEPCs are assumed to have evolved from nonphotosynthetic PEPC [14,15]. The enzymatic differences between C_4_ PEPC and nonphotosynthetic PEPC have been investigated, and characteristic features such as higher tolerance to allosteric inhibitors but low catalytic efficiency, have been observed by analysis of the isoforms. Such comparison among isoforms have been conducted on *Flaveria* [14] and rice [16] enzymes, and the enzymatic differences of each isoform have been determined, leading to the estimation of their functions. However, the relationships between photosynthetic and nonphotosynthetic PEPCs in CAM plants remain unclear, particularly in obligate CAM plants.

Pitaya (*Hylocereus undatus* Britt and Rose) commonly known as dragon fruit, is a subtropical fruit that belongs to the Cactoideae subfamily in the Cactaceae family [17]. Because pitaya can withstand temperatures above −2 °C [18], it can be cultivated in warm temperate regions in unheated plastic greenhouses if an appropriate management system is developed. For this purpose, physiological aspects of pitaya throughout the year in temperate regions should be clarified. As Pitaya undergoes obligate CAM-type photosynthesis [19,20], PEPC plays a crucial role in carbon assimilation throughout the year. Previously, we reported that PEPC activity exhibited a diurnal cycle in summer, but not in winter, in a temperate region (Kobe, Japan) [21]. However, this difference was only the general activity. Because any PTPC consist of multigene family as mentioned above, the number and role of isoforms in pitaya should be determined. To clarify the difference in the role of PEPC isoforms, this study was primarily conducted to identify PEPC isoforms in pitaya. As a result, a novel type of PEPC that lacks a phosphorylation motif was discovered, and it may be a characteristic of Cactaceae plants. This is the first report on, not only the lack a phosphorylation motif isoform of PTPC, but also their complete sequences derived from members of the Cactaceae family.

## 2. Results

### 2.1. Amino Acid Sequence Alignment and Phylogenetic Analysis of PEPC Isoforms

**Figure 1:**
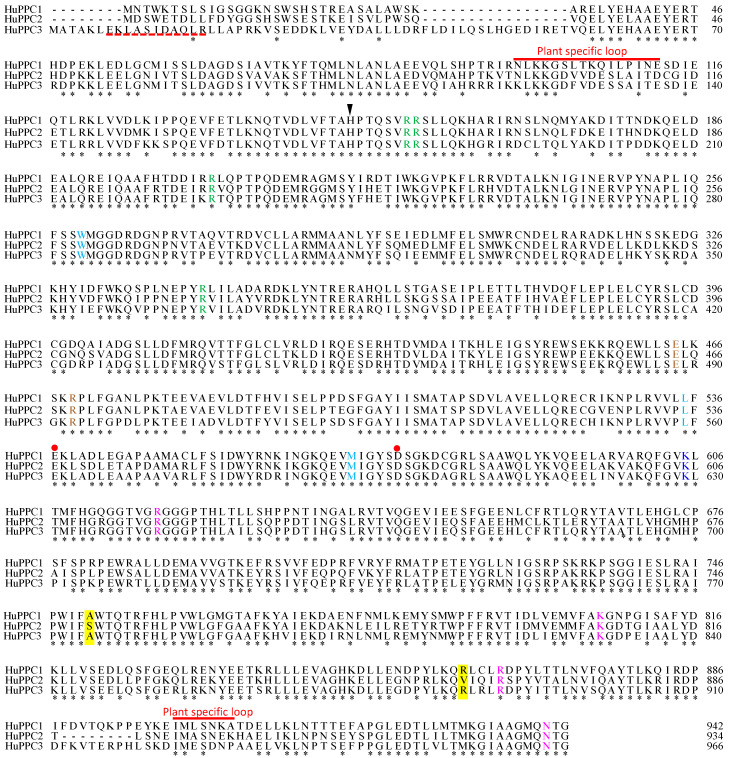
Amino acid sequence alignment of HuPPC1, HuPPC2, and HuPPC3. Asterisks indicate identical amino acid residues in all three proteins. Conserved functional residues are indicated as follows: reverse triangle, catalytic base (H177, in *Zea mays* numbering); red circle, metal binding (E566 and D603, in *Z. mays* numbering); green letter, G-6-P binding (R183, R184, R231, and R372 in *Z. mays* numbering); brown letter, tetramer formation (E493 and R498, in *Z. mays* numbering); magenta letter, Asp binding (R647, K835, R894 and N968 in *Flaveria* numbering); cyan letter, hydrophobic pocket in active site (W248, L504 and M538, in *Z. mays* numbering); navy blue letter, monoubiquitination lysine (K628 in *Ricinus communis* numbering) [22]. Two yellow background portions indicate putative specific substituted amino acids in C_4_ phospho*enol*pyruvate carboxylase. Red broken underline of HuPPC3 represents a phosphorylation motif observed in most PTPCs (acid-base-X-Y-SIDAQLR). Relationships between residue number and function are based on the report by Kai et al. [23].

Three different sequences corresponding to PEPC were obtained using RT-PCR with a degenerate primer pair (PPCF and PPCR). To obtain the full-length cDNA of each isoform, 3′-RACE and 5′-RACE were performed using specific primers for each sequence. Finally, three cDNAs, referred to as *HuPPC1* (accession No. JF966381), *HuPPC2* (accession No. JF966382), *HuPPC3* (accession No. MH104709), respectively, were obtained. *HuPPC1* consisted of 3236 bp with a 2826 bp open reading frame (ORF) encoding 942 amino acid residues, *HuPPC2* consisted of 3318 bp with a 2802 bp ORF encoding 934 amino acids, and *HuPPC3* was 3337 bp in length with a 2898 bp ORF encoding 966 amino acid residues. The deduced amino acid sequences exhibited approximately 78% identity for each isoform combination. Figure 1 shows the multiple sequence alignment of the three isoforms. All amino acids contributing to various PEPC properties, such as catalytic base, malate binding, and G-6-P binding, were conserved in all of the isoforms. However, the crucial difference was that HuPPC1 and HuPPC2 lacked approximately 20 residues in N-terminal region, including Ser-phosphorylation motif, which was observed in other PTPCs.

**Figure 2:**
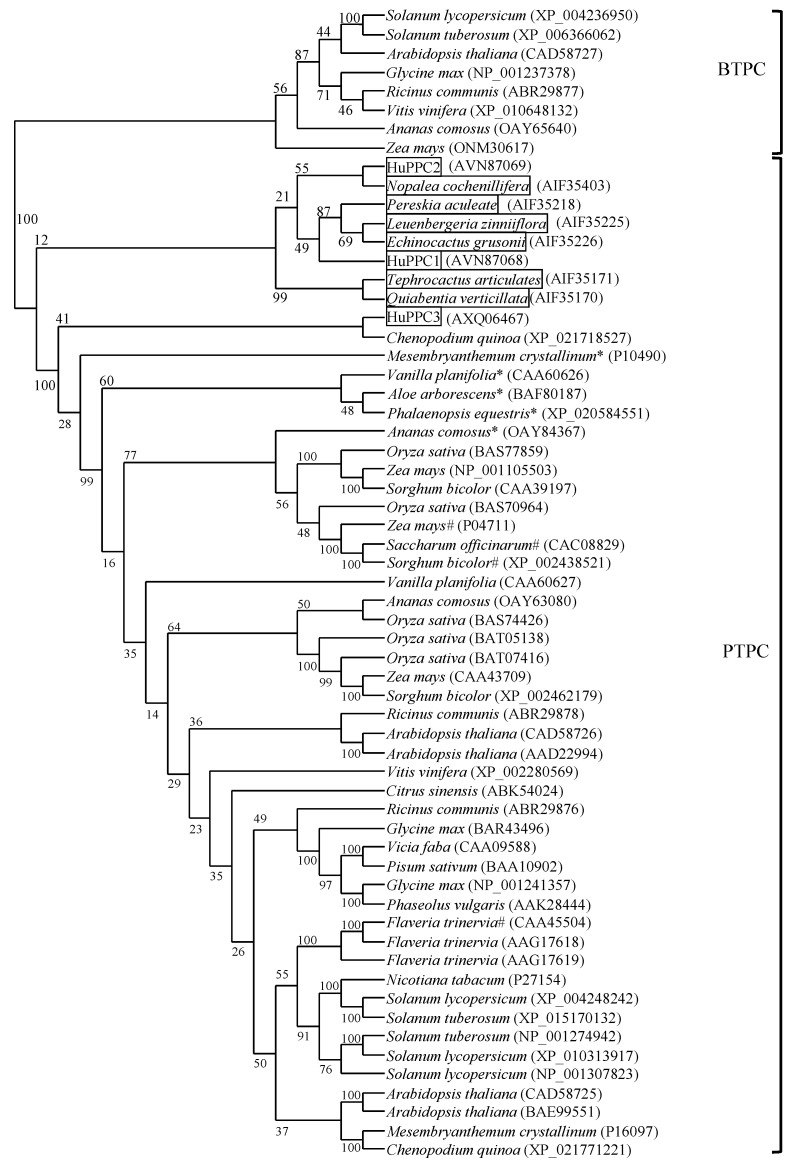
Phylogenetic relationship of 61 phospho*enol*pyruvate carboxylases, including 3 pitaya isoforms from various plants. Values on branches represent bootstrap support (%). Accession numbers are mentioned in parentheses. Asterisk and hash symbols indicate CAM- and C_4_- related isoforms, respectively, and Cactaceae plants were boxed.

Figure 2 shows a phylogenetic tree of 61 PEPCs of various plants including three isoforms found in this study. BTPC and PTPC were obviously separated, and the three pitaya isoforms belonged to PTPC. HuPPC1 and HuPPC2 belonged to a small clade with other PEPCs originating from Cactaceae plants. HuPPC3 was relatively different from these two isoforms and was included in a small clade with other CAM-related PEPCs, except *Chenoposium quinoa*. In contrast, C_4_ plants also belonged to a small clade, except for *Flaveria trinervia*. Because only partial sequences (approximately 500 residues on the C-terminal side) from Cactaceae plants have been registered, we performed analyses using the corresponding C-terminal region in all PEPCs. However, the obtained phylogenetic tree was not significantly different from that shown in Figure 2 (data not shown).

### 2.2. Partial Purification and Characterization of HuPPC Isoforms

**Figure 3:**
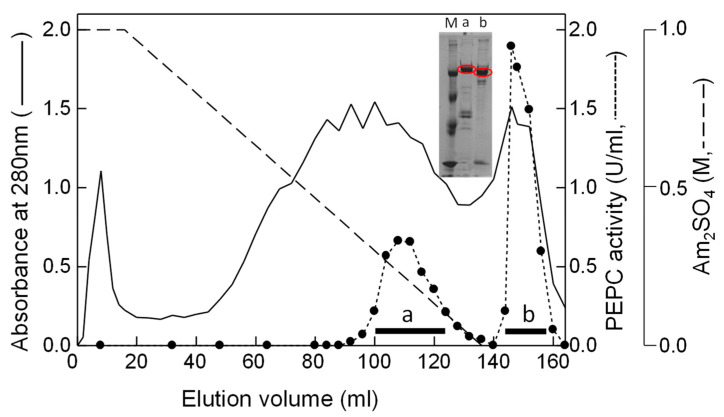
Separation of pitaya phospho*enol*pyruvate carboxylase isoforms on Phenyl-Sepharose column. The solid line, dotted line, and dashed line represent absorbance at 280 nm, PEPC activity, and ammonium sulfate concentration, respectively. The inserted picture is the SDS-PAGE result of the obtained partially purified HuPPCa and HuPPCb (red circled).

To confirm that HuPPC1 and HuPPC2 do not have a phosphorylation motif in N-terminal region, we attempted to purify these isoforms as proteins. Because the pitaya cladode contains large amount of mucilage, the proteins in extracts were absorbed and concentrated through an anion exchange resin with a batch system. Thereafter, two major active fractions were obtained by a Phenyl-Sepharose column chromatography (Figure 3).

Unfortunately, only two isoforms (HuPPCa and HuPPCb), instead of three were partially purified through subsequent runs of column chromatography (Table 1). After SDS-PAGE and transferring the proteins onto a PVDF membrane, the corresponding bands were cut off and taken for protein sequencing. However, no N-terminal sequence was detected for either isoform, probably because both N-termini were blocked. To assign HuPPCa and HuPPCb to their specific isoforms, MASCOT analysis was performed on an in-gel tryptic digest of HuPPCa and HuPPCb. Because an N-terminal region fragment could not be obtained from any of the isoforms, no direct assignment was possible. However, the protein sequence coverage indicates that HuPPCa and HuPPCb corresponded to HuPPC3 and HuPPC2, respectively (Table 2); HuPPC1 was not be obtained as protein. Hereafter HuPPCa and HuPPCb are as expressed HuPPC3 and HuPPC2, respectively.

**Figure 4:**
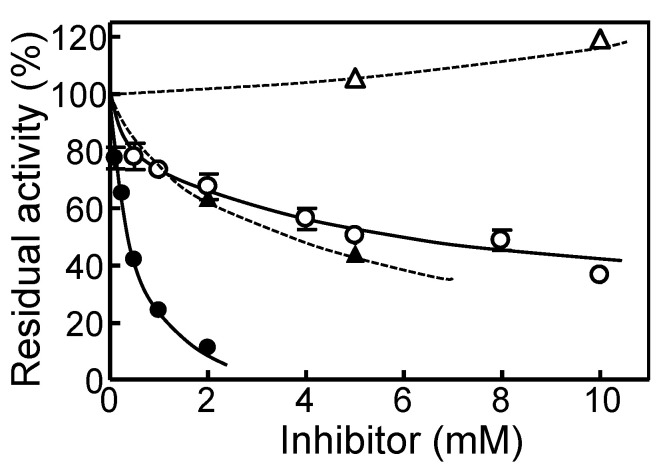
Inhibition on phospho*enol*pyruvate carboxylase activity by malate and Asp. Closed and open symbols represent HuPPC3 and HuPPC2, respectively. Solid lines and circles represent malate inhibition, and dotted lines and triangles represent Asp inhibition.

The basic enzymatic properties of HuPPC3 and HuPPC2 are summarized in Table 3. The *K*_m_ for PEP, pH stability, and optimal pH were not different between HuPPC3 and HuPPC2; however, heat stability and optimal temperature were considerably different. Compared with HuPPC2, HuPPC3 was stable and active at a higher temperature. The inhibitory effects of malate and Asp on PEPC activity were significantly different between HuPPC2 and HuPPC3 (Figure 4). HuPPC3 was inhibited by approximately 90% with 2 mM malate; however, HuPPC3 retained 40% activity with 10mM malate. In the case of Asp, HuPPC3 was inhibited by approximately 50%, whereas HuPPC2 was not inhibited at 10 mM and exhibited an activation trend. Thus, it has been determined that HuPPC3 was more sensitive to malate and Asp than HuPPC2.

To estimate stability, an assay was conducted after the enzyme solution was left to stand for 30 min under the corresponding conditions. The buffer used for pH dependence were MES (pH 5.0–6.5), HEPES (pH 6.5–7.5), and Tris-HCl (pH 7.5–8.5), respectively. Temperature dependence and *K*_m_ measurements were performed at standard conditions described in the Materials and Methods. For evaluation of optimal pH and temperature, the results were corrected based on the stability of malate dehydrogenase under the aforementioned conditions.

## 3. Discussion

Regarding PEPCs in pitaya, using a microarray analysis of drought response, Fan et al. [24] estimated that pitaya has at least three isoforms and one sequence has been registered at NCBI (accession no. AHF21553). However, the sequence of AHF21553 showed only approximately 80% identity with any of the sequences determined in this study. If pitaya has three PEPC isoforms, AHF21553 should exhibit almost 100% identity to at least one of three isoforms determined in the present study. Hence, is AHF21553 a fourth PEPC isoform in pitaya? Multiple sequence alignment of these four sequences indicated that AHF21553 is a mixture of these three isoforms (data not shown). The amino acid sequence of AHF21553 was divided into three regions (N-region, 1-295; M-region, 296-624; and C-region, 625-966), and then the identity of each region to each isoform was recorded. As a result, N-region and HuPPC3, M-region and HuPPC1, and C-region and HuPPC2 exhibited high identities (>95%, Supplementary Materials Table S1). Additionally, we could not find the AHF21553 sequence through RT-PCR using a specific primer set corresponding to N- and C-terminal regions (data not shown). These results suggest that the AHF21553 sequence was probably a disassembled one. Therefore, we conclude that pitaya has at least three PEPC isoforms and their sequences were determined in this study.

Because various important amino acid residues associated with the properties of PEPC, for example, catalytic base (H177 in *Zea mays* numbering) [25], G-6-P binding (R183, R184, R231, and R372 in *Z. mays* numbering) [26], and Asp binding (R647, K835, R894, and N968 in *Z. mays* numbering) [25], are conserved in these three sequences, all isoforms must have PEPC activity and binding ability for allosteric effectors (details are shown in legend of Figure 1). However, the crucial difference is that HuPPC1 and HuPPC2 did not have the Ser-phosphorylation motif in the N-terminal region, which is important to regulate the activity and the sensitivity for allosteric effectors. In CAM photosynthesis, the phosphorylation of the Ser residue catalyzed by PPCK suppresses the inhibitory effects of malate and Asp and enhance the activation by G-6-P at night. During the day, dephosphorylation by PP2A restores the sensitivity to allosteric effectors. The CAM-related PEPC should have the Ser-phosphorylation motif because the circadian rhythm of CAM photosynthesis has been assumed to be a contribution of PPCK [12,13]. Hence, among the three isoforms, HuPPC3 is the one that probably participates in CAM photosynthesis. The phylogenetic analysis also supports this assumption because HuPPC3 belongs to a small clade with other CAM-related PEPCs. What, then, does the lack of a Ser-phosphorylation motif in HuPPC1 and HuPPC2 mean? Chollet et al. [10] reported that deletion of the N-terminal region led to reduced sensitivity for malate, resulting in high activity. This implied that the enzyme lacking the motif was correlated with only low sensitivity for allosteric inhibitors but not for high activity. On the other hand, PEPC is considered to be phosphorylated by PEPCK in all plants [27], and it is said that housekeeping type PEPC also corresponds to the 24-h cycle of PEPCK in *Phalaenopsis* [28]. Thus, HuPPC1 and HuPPC2 have constant activities regardless of phosphorylation or circadian rhythms, and their activities may be regulated by only by the concentration of inhibitors, depending on environmental conditions.

Resistance to allosteric inhibitors is generally higher in C_4_-type than in the C_3_-type, and it is assumed that this property is due to substitution of some amino acid residues [14,29]. According to this criterion, HuPPC1 and HuPPC3 have C_3_-type properties, whereas HuPPC2 is a C_4_-type PEPC even though Arg884 is replaced by Val (Figure 1). In *F. trinervia*, a C_4_ plant, Gly884 contributes to reducing the sensitivity for malate to less than that of C_3_ plants, such as *F. pringlei* which has Arg residue at the corresponding position [29]. In HuPPCs, the corresponding residue is Arg in HuPPC1 (861st) and HuPPC3 (885th), whereas it is Val861 in HuPPC2. The results of malate and Asp inhibition (Figure 4) showed similar trend; that is, HuPPC3 was more sensitive than HuPPC2. Jacobs et al. [30] reported that C-terminal region (region 5, residue 645-966 in *Flaveria* PEPC) is involved in the sensitivity for the malate and Asp inhibitors when *Flaveria* PEPC was divided in five regions. These results suggest that Arg residue (or positive charge) is essential for malate and/or Asp binding, and replacement with other amino acids interferes with their binding, depending on the charge or bulkiness of the side-chain. Furthermore, replacement of Ser774 with Ala in C_3_ *Flaveria* leads to a decreased *K*_m_ value for PEP [31], although Rosnow et al. [32] reported that the corresponding Ser residue did not participate in PEP binding in Chenopodiaceae PEPC. In the results of the present study, the corresponding positions were Ser751 in HuPPC2 and Ala775 in HuPPC3; however, the *K*_m_ values for PEP were not different (Table 3). Recently, DiMario and Cousins [33] reported that the Ser residue is linked to the binding of PEP and CO_3_^−^. Therefore, the Ser residue does not contribute to the PEP binding solely. For PEP binding, Muramatsu et al. [16] has suggested that Lys119 in rice isozyme 2a (corresponding Lys121 in HuPPC3) was conserved in any cytosolic PTPC regardless of the photosynthetic type, and that it contributes to PEP binding together with the charge and length of the junction region. The Lys residue was conserved in any PTPC, but it was replaced with Asn (corresponding to 97th residue) in HuPPC1 and HuPPC2 (Figure 1). This indicates that HuPPC1 and HuPPC2 have PEP binding properties different from those of other cytosolic PEPCs.

The phylogenetic analysis indicated that all isoforms belonged to PTPC, and that HuPPC1 and HuPPC2 consisted of a small clade together with other Cactaceae plant PEPCs. HuPPC3 was somewhat different from these two isoforms but was comparatively closely related with other CAM-type PEPCs, except for *C. quinoa*, which is a C_3_ plant [34]. Among Cactaceae plant PEPCs, excluding those from pitaya, only partial amino acid sequences (approximately 500 residues in the C-terminal side) were registered at NCBI, indicating that the region contains a sequence that exhibits features unique to Cactaceae plants. Of course, any Cactaceae plants PEPC may consist of a multigene family; however, the sequence of only one isoform has been registered. Considering the aforementioned description, the C-terminal region may contribute to the sensitivity to inhibitors, and Cactaceae plants should have inhibition pattern different from those of other plants, resulting in a peculiar regulation profile.

In conclusion, pitaya has at least three PEPC isoforms: HuPPC1, HuPPC2, and HuPPC3. Among them, we assume that HuPPC1 and HuPPC2 are the C_3_-type and C_4_-type, respectively, without regulation by phosphorylation and that HuPPC3 contributes to CAM photosynthesis through circadian oscillation of phosphorylation. Unfortunately, in the present study, HuPPC1 could not be obtained protein. This is probably because HuPPC2 and HuPPC3 are constitutively expressed, whereas HuPPC1 is regulated by various environmental conditions. Both HuPPC1 and HuPPC2 do not have a Ser-phosphorylation motif, but there is an observable difference in sensitivity to inhibitors. Additionally, HuPPC1 and/or HuPPC2 are supposed to have characteristic features unique to Cactaceae plants. To verify these assumptions, it is necessary to analyze the expression profile and enzymatic properties using crystallographic structure imaging and elucidate the complete sequence of PEPCs in other Cactaceae plants.

## 4. Materials and Methods

### 4.1. Plant Material

Cladodes were sampled from 8-year-old white pitayas (*Hylocereus undatus* Britt and Rose) which were cultivated in 50-L pots in an unheated glasshouse at Kobe University, Japan. The cladodes used for enzymes and RNA extraction were sampled on 20 July 2016, at 16:00 (when both enzyme activities were relatively higher than at other times) and stored at −80 °C until used.

### 4.2. Extraction of RNA

Total cellular RNA was prepared from lyophilized pitaya stem. Approximately 1 g of materials was ground in liquid nitrogen and extracted with 10 mL of 0.1 M Tris-HCl buffer (pH8.0) containing 1% CTAB, 0.1 M LiCl, 10 mM EDTA, and 2% (*v*/*v*) 2-mercaptoethanol. Total RNA was purified by the phenol/SDS method previously described by Chirgwin et al. [35].

### 4.3. cDNA Cloning of PEPC

The primers used in the experiment are shown in Supplementary Materials Table S2. First-strand cDNA was synthesized from the total RNA isolated from the stem with ReverTra Ace (TOYOBO Co. Ltd., Osaka, Japan) using an adaptor primer for 3′-RACE at 42 °C for 1 h. The cDNA product was then subjected to PCR amplification with KOD-plus-NEO (TOYOBO Co. Ltd.) using a primer set of PEPCF and PEPCR. The PCR amplified fragment (1800 bp) was cloned into pTA2 vector using TArget Clone^™^-Plus-(TOYOBO Co. Ltd.) and sequenced. The sequence of a fragment was determined using a BigDye Terminator Cycle Sequencing Kit (Applied Biosystems, Waltham, MA, USA), and the fragment was sequenced in an ABI 3130xl Genetic Analyzer (Applied Biosystems). For subsequent RACE experiments, primers were designed based on the fragments’ sequences obtained previous step, using a GeneRacer kit (Invitrogen, Waltham, MA, USA). The primers used for the 5′-RACE were insert used for reveres transcription, for the first round, and for the nested PCR. The primers used for the 3′-RACE were adaptor primers for reverse transcription, for the first round, and for the nested PCR. In both cases, the final products were cloned into pTA2 vector using TArget Clone^™^-Plus-(TOYOBO Co. Ltd.) and sequenced.

### 4.4. Phylogenetic Analysis

Regarding 61 PEPCs including 3 pitaya isoforms, multiple sequence alignment, and construction of a phylogenetic tree using the Neighbor-Joining method [36] were carried out using Seaview version 4.3.3 software (http://pbil.univ-lyon1.fr/software/seaview.html). Default parameters for PhyML tree were used, except for 1000 bootstrap replicates. Amino acid sequences were obtained from the NCBI data base. For six Cactaceae plants, approximately 500 residues on the C-terminal side were used, because no complete sequence was registered.

### 4.5. Partial Purification of HuPPC

#### 4.5.1. Extraction and Concentration Using Anion Exchange Resin

The lyophilized cladodes were homogenized using 20 volumes of extraction buffer (0.1 M Tris-HCl buffer at pH 7.5 containing 10 mM 2-mercaptoethanol, 1 mM EDTA, 10 mM MnCl_2_, 5% (*w*/*v*) PEG6000, and protease inhibitor cocktail (Nacalai Tesque). The homogenate was centrifuged at 23,000× *g*, for 20 min at 4 °C, and 40 mL of a Q-Sepharose Fast Flow (GE Healthcare Japan) equilibrated with 0.1 M Tris-HCl buffer at pH 7.5 containing 10 mM 2-mercaptoethanol was added to the supernatant. After stirring, the Q-Sepharose resin was collected using glass filter (3G) and this step was repeated three times. Finally, the resin was packed in the column (*ϕ*2.5 × 9 cm) and washed with a 10-fold volume of the same buffer. PEPC activity was evaluated by eluting it with the same buffer containing 0.3 M NaCl.

#### 4.5.2. Phenyl-Sepharose Column Chromatography

After salting-out by 60% saturation ammonium sulfate of the obtained fraction having a PEPC activity, the collected pellet was dissolved in an aliquot of 20 mM Tris-HCl buffer at pH 8.0 containing 1 M ammonium sulfate and 10 mM 2-mercaptoethanol and then applied on a Phenyl-Sepharose column (*ϕ*1.5 × 4 cm) equilibrated with the same buffer. After washing with a 10-fold volume of the same buffer, the enzymes were eluted by gradient elution with an ammonium sulfate concentration from 1 to 0 M (50 mL each). In this step, two active fractions were obtained: HuPPCa and HuPPCb. The subsequent column chromatographies were performed using the FPLC systems.

#### 4.5.3. Mono Q Column Chromatography

Both PEPC fractions obtained were dialyzed against 50 mM Tris-HCl buffer at pH 8.0 containing 10 mM 2-mercaptoethanol and then applied on a Mono Q column equilibrated with the same buffer and eluted with a linear NaCl concentration gradient.

#### 4.5.4. Hydroxyapatite (HA) Column Chromatography

The active fraction that was obtained using the Mono Q column was applied on a HA column equilibrated with 15 mM phosphate buffer at pH 7.0 containing 10 mM 2-mercaptoethanol. Elution was carried out with a linear concentration gradient of phosphate to 150 mM.

#### 4.5.5. Mono S Column Chromatography

The obtained active fraction was applied on a Mono S column equilibrated with 50 mM MES buffer at pH 5.8 containing 10 mM 2-mercaptoethanol. Elution was carried out with a linear NaCl concentration gradient.

The protein content was measured using the modified Lowry method [37].

### 4.6. Enzyme Assay

PEPC activity was spectrophotometrically measured by evaluating the absorbance change at 340 nm caused by the oxidation of NADH to NAD at 30 °C. The standard 1 mL assay mixture consisted of 0.1 M HEPES buffer at pH 7.0 containing 10 mM 2-mercaptoethanol, 1 mM EDTA, 10 mM MgCl_2_, 0.2 mM NADH, 2 mM PEP, 10 mM NaHCO_3_, and 3 U of malate dehydrogenase (TOYOBO). One unit of enzyme activity was defined as the amount catalyzing the change of 1 μmol of NADH per minute. In the inhibition assay, a standard system was used, and the concentration of the inhibitor was sequentially changed.

### 4.7. MS Analysis to Identify the Amino Acid Sequence

SDS-PAGE was carried out according to the method of Laemmli [38] using 10% gel. After CBB staining, bands corresponding to HuPPCa and HuPPCb were excised from the gel and assigned to the corresponding amino acid sequences. Peptide mass fingerprinting of the trypsin-digested peptides was performed at the Center for Supports to Research and Education Activities at Kobe University. For the MS analysis, trypsin-digested peptides were separated by Paradigm MS2/HTS-PAL HPLC (Michrom BioResources Inc., Auburn, CA, USA) and analyzed on an LTQ-orbitrap Discovery (Thermo Fisher Scientific). For the MS data, *m*/*z* values were queried against the amino acid sequences of HuPPC1, HuPPC2, and HuPPC3 to assign each polypeptide sequence using the MASCOT algorithm (Matrix Science, Boston, MA, USA).

## Figures and Tables

**Table 1:** Summary of purification of phospho*enol*pyruvate carboxylase from pitaya cladode.

	Total Activity (U)	Recovery (%)	Specific Activity (U/mg)	Total Protein (mg)	Purification Fold
Extract	33.3	100	0.0318	1050	1
Q-Sepharose	24.3	73.0	0.122	199	3.84
Phenyl-Sepharose-a	9.10	27.4	0.315	28.9	9.91
Phenyl-Sepharose-b	11.3	34.1	0.486	23.3	15.3
Mono Q-a	7.18	21.6	2.19	3.28	68.9
Mono Q-b	8.17	24.6	0.836	9.78	26.3
Hydroxyapatite-a	4.37	13.1	3.87	1.13	122
Hydroxyapatite-b	7.43	22.3	1.49	5.00	46.9
Mono S-a	4.24	12.7	7.45	0.256	234
Mono S-b	1.91	5.73	1.07	2.38	33.6

Small letters, a and b, represent the fractions a and b in Figure 3, respectively.

**Table 2:** Sequence coverage of HuPPCa and HuPPCb to each isoform.

	HuPPC1	HuPPC2	HuPPC3
HuPPCa	12%	18%	75%
HuPPCb	10%	67%	13%

**Table 3:** Physicochemical properties and pH dependence of *K*_m_ values for phospho*enol*pyruvate.

	HuPPC2	HuPPC3
pH stability	6.5–7.0	5.5–6.5
Heat stability	Up to 35 °C	Up to 50 °C
Optimal pH	6.5–7.2	7.0–7.2
Optimal temperature	28 °C	55 °C
*K*_m,PEP_ (μM)	44.3 ± 7.0	48.5 ± 4.0

## Supplementary Materials

The following are available online at https://www.mdpi.com/2223-7747/9/9/1241/s1, Table S1: Sequence identity of each isoform to AHF21553. Table S2: Primer’s sequences used in this study.
