# Supplementary material for: Molecular Cloning of Novel-Type Phosphoenolpyruvate Carboxylase Isoforms in Pitaya (Hylocereus undatus)
Source: Plants (Basel). 2020 Sep 21;9(9):1241. doi: 10.3390/plants9091241 (PMC7569800; doi:10.3390/plants9091241)
Supplement: Supplementary file 1 [file plants-09-01241-s001.pdf]

Table S1. Sequence identity of each isoform to AHF21553.

	Region of AHF21553		
	N-region	M-region	C-region
HuPPC1	72 %	95 %	70 %
HuPPC2	72 %	82 %	98 %
HuPPC3	99 %	82 %	77 %

N-, M- and C-region indicate the amino acid residues of AHF21553 1-295, 296-626 and 625-966, respectively.

Table S2. Primer's sequences used in this study.

Primer	Sequence	Purpose
PEPCF	5'-TTYTCNTCNTGGATGGGNGG-3'	RT-PCR(FSSWM)
PEPCR	5'-TGRAAYCTNTGYTGNTGCCA-3'	RT-PCR(WTQTR)
RT primer	5'-GGCCACGCGTCGACTAGTAC$(T)_{17}$-3'	RT, 3'-RACE
adaptor	5'-GGCCACGCGTCGACTAGTAC -3'	3'-RACE
P1(3'CPSFfw)	5'-TGTCCATCTTTCTCACCTAGG-3'	3'-RACE
P2(3'HPAIfw)	5'-CATCCCGCGATCTCTCCACTCC-3'	3'-RACE
P3(3'HPPIfw)	5'-CACCCACCTATCTCTCCTAAGC-3'	3'-RACE
P1(3'DVTQfw)	5'-GATGTAACACAGAAACCACCAG-3'	3'-RACE sequencing
P2(3'QVIQfw)	5'-AACAGGTTATCCAAATTCGCAG-3'	3'-RACE sequencing
P3(3'KVTEfw)	5'-CAAGGTAACAGAGAGGCCTCA-3'	3'-RACE sequencing
DVLAV:rev	5'-AAGCTCAACAGCAAGCACAT-3'	5'-RACE RT
P1(5'CLVRrv)	5'-TAATATCAAGTCTTACGAGGC-3'	5'-RACE 1st nested
P2(5'CLTKrv)	5'-TTATGTCAAGCTTCGTGAGAC-3'	5'-RACE 1st nested
P3(5'SLVRrv)	5'-TGATATCAAGCCTCACAAGAG-3'	5'-RACE 1st nested
P1(5'SEIPrv)	5'-TCTCCAAAGGGATTTCGGAGG -3'	5'-RACE 2nd nested
P2(5'CLLArv)	5'-ATCATTCTGGCCAGTAAGCA-3'	5'-RACE 2nd nested
P3(5'SDIPrv)	5'-CTTCCTCAGGGATATCAGAGA -3'	5'-RACE 2nd nested
P1(Nterm)	5'-GATGAACACCTGGAAAACCAG-3'	5'-RACE sequencing
P2(Nterm)	5'-AATGGACAGTTGGGAAACCGA-3'	5'-RACE sequencing
P3(Nterm)	5'-ATGGCGACTGCCAAGTTGGAGAA -3'	5'-RACE sequencing
AHF21553fw	5'-ATGGCGACTGCCAAGTTAGAGAAATTGG-3'	RT-PCR of AHF21553
AHF21553rv	5'-CTAACCAGTGTTCTGCATGCCAGCAGCA-3'	RT-PCR of AHF21553
